# Thermally Stimulated Currents in Nanocrystalline Titania

**DOI:** 10.3390/nano8010013

**Published:** 2018-01-05

**Authors:** Mara Bruzzi, Riccardo Mori, Andrea Baldi, Ennio Antonio Carnevale, Alessandro Cavallaro, Monica Scaringella

**Affiliations:** 1 Dipartimento di Fisica e Astronomia, Università di Firenze, Via G. Sansone 1, Sesto Fiorentino, 50019 Firenze, Italy; 2 Albert-Ludwigs-Universität Freiburg, Experimentelle Teilchenphysik, Physikalisches Institut, Hermann-Herder Straße 3, 79104 Freiburg im Breisgau, Germany; riccardo.mori@physik.uni-freiburg.de; 3 Dipartimento di Ingegneria Industriale, Università di Firenze, Via S. Marta 3, 50139 Firenze, Italy; andrea.baldi@unifi.it (A.B.); ennio.carnevale@unifi.it (E.A.C.); 4 LBT Observatory, University of Arizona, 933 N. Cherry Ave, Tucson, AZ 85721, USA; cavallaro@lbto.org; 5 Dipartimento di Ingegneria dell’Informazione, Università di Firenze, Via S. Marta 3, 50139 Firenze, Italy; monica.scaringella@unifi.it

**Keywords:** thermally stimulated currents, photocurrent, titanium dioxide, hopping, nanoporous film, desorption current, chemisorbed current

## Abstract

A thorough study on the distribution of defect-related active energy levels has been performed on nanocrystalline TiO_2_. Films have been deposited on thick-alumina printed circuit boards equipped with electrical contacts, heater and temperature sensors, to carry out a detailed thermally stimulated currents analysis on a wide temperature range (5–630 K), in view to evidence contributions from shallow to deep energy levels within the gap. Data have been processed by numerically modelling electrical transport. The model considers both free and hopping contribution to conduction, a density of states characterized by an exponential tail of localized states below the conduction band and the convolution of standard Thermally Stimulated Currents (TSC) emissions with gaussian distributions to take into account the variability in energy due to local perturbations in the highly disordered network. Results show that in the low temperature range, up to 200 K, hopping within the exponential band tail represents the main contribution to electrical conduction. Above room temperature, electrical conduction is dominated by free carriers contribution and by emissions from deep energy levels, with a defect density ranging within 10^14^–10^18^ cm^−3^, associated with physio- and chemi-sorbed water vapour, OH groups and to oxygen vacancies.

## 1. Introduction

The study of the electronic transport in nanocrystalline Titanium dioxide (nc-TiO_2_) is motivated by its wide range of application, from catalysis to green energy systems such as Dye Sensitized Solar Cells (DSSCs) [1] and toxic gas sensing devices [2]. In fact, performances of devices based on nc-TiO_2_ strongly depend on electron transport mechanisms, which can be very different from those dominating in the bulk single-crystal semiconductor, due to the complex morphology structure and to the huge active surface of this porous material with respect to its volume. Investigation of the electrical transport properties of nc-TiO_2_ and its relationship with surface and bulk defects is thus strategic in the perspective of increasing their performance. A model of the electrical conductivity in nc-TiO_2_ that takes into account all the complexities of this material (disorder, fractional dimensionality of the nanoporous material and potential barriers between the constituent nanoparticles) is still lacking; its implementation is a cumbersome task. In order to progress in this direction, a systematic characterisation of defects states, in the widest range of energy and an evaluation of their concentration in the material is mandatory.

The thermally stimulated current technique is one of the most effective tool for characterizing electrical defects in semiconductors. In this paper, we apply this method to get an overall picture of defect distribution in nanocrystalline Titania used in state-of-art DSSC devices. For ordinary semiconductors the well established models of the Thermally Stimulated Currents (TSC) give a quantitative description of the experimental results and then allow the extraction of the parameter values; for disordered semiconductors in general, and more specifically for nanocrystalline mesoporous materials, only a few studies are available and, as a consequence of their complexity, of difficult implementation for a numerical fit of the experimental data [3]. These latter give evidence that TiO_2_ nanoparticles have a great number of bulk and surface defects inducing a structural and energetic disorder, and, as a consequence, a continuum of levels into the band gap [4]; but the experimental frame is even more complex because when a film is deposited, the nanoparticles give rise to a porous network, with a porosity typically ranging between 0.5% and 0.7% [5], where a large amount of them are separated by potential barriers [6]. A theory of the electrical conduction in disordered and nanocrystalline materials is the basis for a model of the conductivity in the mesoporous TiO_2_ [7] and several studies have been devoted to establish if the hopping or multi-trapping mechanism prevails. The conduction in the hopping mechanism is given by the carrier tunneling between different sites, and in the multi-trapping mechanism by a series of successive events where the carriers are temporarily promoted from localized levels to the conduction band; unfortunately the experimental signatures of the two mechanisms are indistinguishable and then what is the prevailing mechanism is still debated [8,9,10,11]. To take into account the porosity of the medium or the interparticle barrier get more complex the theoretical description of the conductivity and we must refer ourselves to models of a disordered medium of fractional dimensionality, i.e., intermediate between 2D and 3D, or to models of a network of perfect nanoparticles; specifically as far as TiO_2_ is concerned, to our knowledge, these aspects have been considered only at a qualitative level [5]. In consideration of the above sketched complexities some strong simplification is necessary in order to extract quantitative information concerning the carrier transport, and specifically the TSC.

The model of Thermally Stimulated Currents (TSC) used to interpret our experimental results, takes account of the heavily disordered microscopic nature of nc-TiO_2_. In this work, we adopt the model usually considered for electrical transport in nc-TiO_2_ which treats this material as a strongly disordered 3D medium and neglects the potential barriers at the grain interface [8,12]; this, despite the strong simplification that it implies, is able to give reason of the main features of the electrical transport and is simple enough to allow the implementation of a numerical algorithm useful for fitting the experimental data. In our model we avoid any a priori assumption about the conduction mechanism, and this is essential because, as we will show, in general both the hopping and free carrier, i.e., the multitrapping, contributions must be taken into account for the electrical conductivity [13]: the possible prevalence of the one or the other depends on the temperature and the Fermi level position. Developed from reference models given in [13,14,15] and first applied in [16] to get an insight about the prevalence of hopping against multitrapping conductivity in nanocrystalline titania below 200 K, in this paper the model has been further extended to determine main parameters of the band tail, as energy decay constant, defect and recombination centres concentrations, hopping frequency factors. Moreover, our results, extended up to 630 K, show that hopping conductivity can be neglected over 200 K, where conductivity appears to be dominated by discrete energy levels in the forbidden gap, probably related to water, OH groups and oxygen vacancies, which role in transport properties is nowadays still a matter of intense debate [17,18].

## 2. Mixed Conduction Model in nc-TiO_2_

In heavily disordered semiconductors, significant carrier hopping can occur between localized sites, whose energies fall within the band gap, giving a non-negligible contribution to the electrical conductivity. As a result, mixed conduction mechanisms with hopping and free carriers contributions should be taken into account when discussing the electrical transport in such a material, especially at low-to-moderate temperatures and high carrier densities.

**Figure 1:**
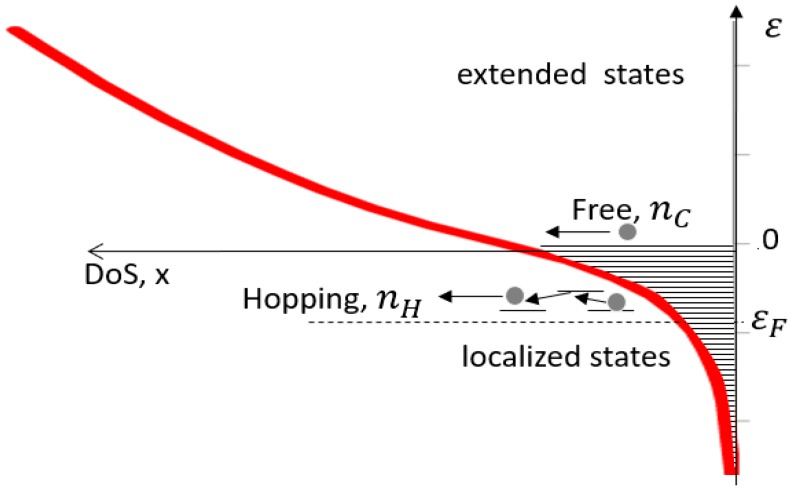
Density of states (DoS) as obtained with an exponential decay tail in the forbidden gap and a square root dependence in the conduction band region. The boundary is kept at $\varepsilon_{C}$ = 0, minimum of the conduction band. Conduction is due to both free electrons in the conduction band and to hopping at localized states.

As it is usually done, we consider the nc-TiO_2_ as a strongly disordered intrinsic semiconductor whose charge carriers are electrons: then the Density of States (DoS) is modelled in the simplest way as the sum of the extended state contribution (i.e., the conduction band), with the typical square root dependence on energy, and the localized state contribution characterized by an exponential tail, below the conduction band, as for amorphous materials [19]. In fact, this model neglects the DoS features in the intermediate region connecting the exponential Urbach tail and the conduction band, whose description has been object of several studies [20]. Such expressions are rather complex and their use, even at the cost of the computation time, do not provide substantial improvements to the fitting procedure. Moreover, in our measurements the Fermi level never attaints this region and the hopping, as well the multi-trapping, take place over an extended part of the DoS, then coming rather insensitive to the DoS details. Then, the density of states in the overall energy range, putting at *ε* = 0 the border between extended and localized states, is given by the function:

(1) $$g\left(\varepsilon \right)=\frac{N_{L}}{E_{L}}\theta \left(-\varepsilon \right)e^{\frac{\varepsilon }{E_{L}}}+\theta \left(\varepsilon \right)N_{C0}\sqrt{\varepsilon +E_{B}},$$

with θ(ε) Heaviside function, *E*_L_ tailing factor, $N_{L}=\int_{-\infty }^{0}g\left(\varepsilon \right)d\varepsilon$ total density of localized states within the band gap. $N_{C0}$ is related to the effective density of states at the conduction band minimum. The value $N_{C0}$ = 4 × 10^19^ cm^−3^ · meV^−3/2^, used for all subsequent calculations, has been chosen in order to get at room temperature $N_{C}$ = 5.2 × 10^21^ cm^−3^, typical for TiO_2_. The energy parameter:

(2) $$E_{B}={\left(\frac{N_{L}}{N_{C0}E_{L}}\right)}^{2},$$

is introduced so as to have the continuity of g(ε) at ε = 0. The DoS obtained with this procedure is shown in Figure 1.

Mixed electrical conductivity, in case of a *n*-type nc-TiO_2_ layer, is the result of transport processes of both hopping electrons within the band tail and free electrons in the conduction band, as depicted in Figure 1, then for the total conductivity we have:

(3) $$\sigma =\sigma_{H}+\sigma_{C}=q\mu_{H}n_{H}+q\mu_{C}n_{C},$$

where $\sigma_{H}$ and $\sigma_{C}$, $\mu_{H}$ and $\mu_{C}$, $n_{H}$ and $n_{C}$, are conductivity, mobility and electron concentration of hopping carriers and free carriers respectively; q is the electron charge. In (3) $\mu_{H}$ is an effective mobility that must be calculated taking into account that the hopping probability depends on the site. Assuming the validity of the Einstein relation, the hopping mobility is related to the carrier energy through the diffusivity D(ε):

(4) $$\mu \left(\varepsilon \right)=\frac{q}{K_{B}T}D\left(\varepsilon \right)=\frac{q}{K_{B}T}r{\left(\varepsilon \right)}^{2}\nu ,$$

with *ν* hopping rate and r(ε) average distance between hopping sites available for hopping for carrier with energy *ε*. The rate for hopping from site i to site j, respectively characterized by energies *ε*_i_ and *ε*_j_, is given by the Miller-Abrahams [21] model:

(5) $$\begin{array}{cc} \nu =\nu_{0}e^{-2\frac{r_{\mathrm{ij}}}{\alpha }-\frac{\varepsilon_{j}-\varepsilon_{i}}{K_{B}T}}, & \varepsilon_{i}<\varepsilon_{j}\\ \nu =\nu_{0}e^{-2\frac{r_{\mathrm{ij}}}{\alpha }}, & \varepsilon_{i}>\varepsilon_{j} \end{array}$$

where $r_{\mathrm{ij}}$ is the distance between sites at $\varepsilon_{i}$ and at $\varepsilon_{j}$, α is the localization radius of the electron and $\nu_{0}$ is the hopping frequency coefficient.

The localization radius can be assumed as a constant, of the order of a few angstrom, for states deeply localized, but when approaching the boundary ε = 0 with the extended states this length diverges. Then, as the hopping probability depends exponentially on this length, the description of the conduction for carriers localized in states near the mobility edge must take into account this behavior. This divergence, according to several models, behaves as $\varepsilon^{-\gamma }$ with γ ranging between 0.5 and 1.5 [19]. Here, we assume for the localization length α:

(6) $$\alpha \left(\varepsilon \right)=\alpha_{0}{\left(1-\frac{z}{\varepsilon }\right)}^{\gamma },$$

with *α*_0_ carrier localization length of the deep states and z the energy to which *α* starts to diverge. The evaluation of the hopping conductivity requires some kind of approximation in order to get an expression useful for fitting experimental data. A considerable simplification is usually obtained referring to the “transport energy” level [8,14,22], that plays for hopping the same role of the mobility edge for the free carriers, but for a quantitave description of the TSC is necessary to go beyond this approach [13]. Then, to this purpose, we will follow the approach proposed by Nagy [15] where the hopping conductivity can be calculated as:

(7) $$\sigma_{H}\left(T\right)=q\int \mu \;dn=\nu_{0}\frac{q^{2}}{K_{B}T}\int_{-\infty }^{0}\;g\left(\varepsilon \right)f\left(e,E_{F},T\right)r{\left(\varepsilon \right)}^{2}e^{-2\frac{r\left(\varepsilon \right)}{\alpha \left(\varepsilon \right)}}d\varepsilon ,$$

where r(ε) is an average hopping distance for a site of energy *ε*, defined in term of the concentration of the available (unoccupied) final states:

(8) $$\begin{array}{c} \frac{1}{r{\left(\varepsilon \right)}^{3}}=\frac{4\pi }{3BR_{L}^{3}}\left(\int_{-\infty }^{\varepsilon }\frac{g\left(\varepsilon^{\prime }\right)}{N_{L}}\left[1-f\left(\varepsilon^{\prime },E_{F},T\right)\right]d\varepsilon^{\prime }+\int_{\varepsilon }^{0}\frac{g\left(\varepsilon^{\prime }\right)}{N_{L}}\left[1-f\left(\varepsilon^{\prime },E_{F},T\right)\right]e^{\frac{\varepsilon -\varepsilon^{\prime }}{KT}}d\varepsilon^{\prime }\right),\\ \frac{1}{R_{L}{}^{3}}=\frac{4\pi }{3}\int_{-\infty }^{0}g\left(\varepsilon^{\prime }\right)d\varepsilon^{\prime }=\frac{4\pi }{3}N_{L} \end{array}$$

$$f\left(\varepsilon ,E_{F},\;T\right)=1-e^{\frac{\varepsilon -\varepsilon_{F}}{KT}}+\frac{1}{2}e^{\frac{3\left(\varepsilon -\varepsilon_{F}\right)}{2KT}}\;\varepsilon \leq \varepsilon_{F}$$

$$f\left(\varepsilon ,E_{F},\;T\right)=e^{-\frac{\varepsilon -\varepsilon_{F}}{KT}}-\frac{1}{2}e^{-\frac{3\left(\varepsilon -\varepsilon_{F}\right)}{2KT}}\;\varepsilon \geq \varepsilon_{F}$$

with $f\left(\varepsilon ,E_{F},T\right)$, Fermi-Dirac distribution function, *α* given by Equation (6) and *B* the percolative limit factor, namely the average number of site links, a parameter dependent on the system dimensionality (*B* = 2.7 for 3D hopping [14]). The integral in Equation (8) is splitted in two terms the first one taking into account, for the level of energy ε , the number of sites reachable with a down conversion process, having probability 1, the second the number of the sites reachable with a thermally activated up conversion process whose probability is given by the Boltzmann factor.

The Fermi energy $E_{F}$ is obtained by evaluating the total electron concentration *n* as the sum of the electron density in the band tail (*ε* < 0), $n_{H}$, and in the conduction band (*ε* > 0), $n_{C}$; then $n=n_{C}+n_{H}$, where:

(9) $$n_{H}=\int_{-\infty }^{0}g\left(\varepsilon \right)f\left(\varepsilon ,E_{F},\;T\right)d\varepsilon ,\;\;\;n_{C}=\int_{0}^{\infty }g\left(\varepsilon \right)f\left(\varepsilon ,E_{F},\;T\right)d\varepsilon ,$$

Finally, the contribution of free electrons to the electrical conductivity is given by the typical expression of the conductivity for electrons in the conduction band:

(10) $$\sigma_{C}\left(T\right)=q\mu_{C}n_{C}=q\frac{\mu_{C0}}{{\left(K_{B}T\right)}^{3/2}}n_{C},$$

with $n_{C}$ as calculated from Equation (9). A typical value for the TiO_2_ mobility constant is $\mu_{C0}$ = 1 cm^2^ meV^3/2^/Vs, which gives a room temperature mobility $\mu_{C}$ = 7.5 × 10^−3^ cm^2^/Vs [23].

## 3. Rate Equations for Priming and Thermally Stimulated Process

The rate equation for hopping and free carriers is:

(11) $$\frac{dn}{dt}=-\left(\frac{dn_{H}}{dt}\right)-\frac{n_{C}}{\tau_{C}}+S\left(t\right),$$

where S(t) is the generation rate during priming (e.g., light exposure, null during the TSC scan). Here conduction carriers decay is considered via annihilation on recombination centers or trapping from deep levels, characterized by an active energy level within the gap. The free electron lifetime, $\tau_{C}$, is typically dependent on the defect capture cross section $\sigma_{t}$ and concentration $N_{t}$:

(12) $$\frac{1}{\tau_{C}}=N_{t}\sigma_{t}\langle v_{\mathrm{th}}\rangle =N_{t}\sigma_{t}\sqrt{\frac{3K_{B}T}{m}},$$

where *m* is the effective mass of the free carriers (in TiO_2_ it is about 7 times the electron mass $m_{0}$ [24]) and $\langle v_{\mathrm{th}}\rangle$ their average thermal velocity. In general, in a disordered semiconductor, defects may have a spread in energy, so its concentration is calculated through a gaussian distribution:

(13) $$N_{t}=\frac{N_{t0}}{\sqrt{2\pi }\sigma_{E_{t}}}\int e^{-\frac{{\left(\varepsilon -E_{t}\right)}^{2}}{2\sigma_{E_{t}}{}^{2}}}d\varepsilon$$

The decay of the hopping carriers, in turn, is given as [14]:

(14) $${\left(\frac{dn_{H}}{dt}\right)}_{dec}=-\int_{-\infty }^{0}\frac{g\left(\varepsilon \right)f\left(\varepsilon \right)}{\tau \left(T,\varepsilon \right)}d\varepsilon ,$$

(15) $$\frac{1}{\tau \left(T,\varepsilon \right)}=N_{t}\frac{r{\left(\varepsilon \right)}^{2}D\left(\varepsilon \right)}{\alpha }=\nu_{0}N_{t}\frac{r{\left(\varepsilon \right)}^{4}}{\alpha }e^{-2\frac{r\left(\varepsilon \right)}{\alpha }},$$

In the following, we will describe results of decayed/fractionated Thermally Stimulated Currents (TSC) experiments [25] analyzed with the mixed conduction model described above. In this method, after priming the sample only once at a low temperature, successive cycles of heating/cooling are applied to fractionally deplete levels of lowering energy. The sample is heated up to a first maximum temperature $T_{\mathrm{stop}}$ and cooled down to a first minimum temperature $T_{\mathrm{start}}$ then it is heated and cooled again to higher T values and so on, eventually up to the final temperature.

At first, we have used the decayed TSC method to get useful information about the DoS shape. For each scan of the decayed TSC, we extracted the couple ($E_{\mathrm{act}}$, Q) with $E_{\mathrm{act}}$ = activation energy as determined from the initial rise of the *TSC* and Q = emitted charge calculated by integrating the TSC of the corresponding scan [25]. In this way, we obtained the DoS shape as a function of the energy by plotting the charge released at each step as a function of the activation energy $E_{\mathrm{act}}$.

Then, delayed TSC measurements have been best fitted using the mixed conduction model, considering a constant heating/cooling rate: $\beta =\frac{dT}{dt}$, starting from the initial condition due to priming, stated that in between two scans there is a time delay in which the electrical state of the sample evolve very slowly. So, assuming the initial state got from priming, it is possible to fit the entire sequence of the delayed TSC at once. The rate equation is solved starting from an evaluation of the Fermi level, then the distance between hopping sites, the hopping carrier density and the average hopping rate are obtained and finally iterating for each temperature the calculation is performed for the entire experimental data set.

In the high temperature range, above room temperature, when conductivity is mainly due to free carriers and hopping can be neglected, in our model TSC is considered as dominated by deep centers with discrete energy levels $E_{t}$ in the forbidden gap, characterized by a capture cross section $\sigma_{n}$ (for electrons) and a trap $N_{t}$ concentration. So, we consider the standard TSC expression as [26]:

(16) $$I_{\mathrm{TSC}}\left(T\right)=q\mu_{C}\left(T\right)\sum N_{t}Fe_{n}\left(T\right)e^{-\frac{1}{\beta }\int_{T_{i}}^{T}e_{n}\left(T\right)dT},$$

with $e_{n}\left(T\right)=N_{C}\sigma_{n}v_{\mathrm{th}}e^{-\frac{E_{C}-E_{t}}{K_{B}T}}$ emission constant, Σ surface normal to electric field *F*, *T*_i_ initial temperature of the scan. Due to the disorder in the nanocrystalline material, the TSC peak usually results in a peak broader in temperature than the standard one. This is due to the fact that the energy $E_{t}$ of a defect varies within a certain range due to local morphological changes. We have taken into account that by convoluting the TSC peak with a gaussian distribution, as given in Equation (13).

## 4. Experimental Set-Up and Procedure

**Figure 2:**
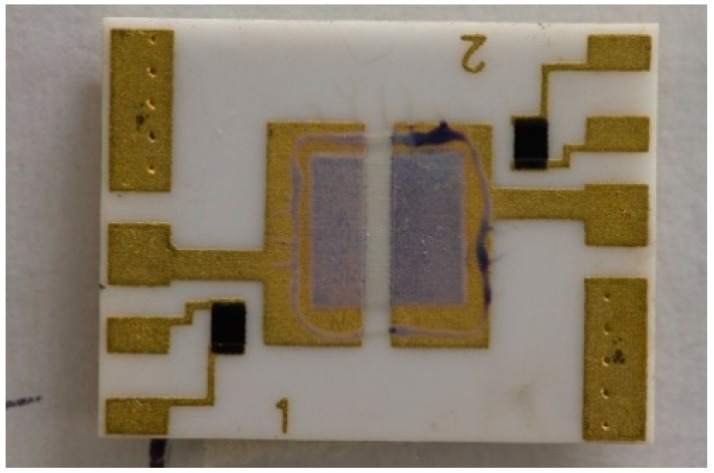
Nc-TiO_2_ film deposited on an alumina chip for Thermally Stimulated Currents (TSC) measurements.

To manufacture our samples, we used a colloidal system produced by Solaronix (Aubonne, Switzerland), containing about 11 wt %. nanocrystalline titanium dioxide mixed with optically dispersing anatase particles (13/400 nm, Ti-Nanoxide D/SP). This commercial product, specifically developed for prototypal electrodes in DSSCs is an anatase titania particle paste for the deposition of active opaque layers. A mixing of large, 400 nm average size, and small, 13 nm average size, nano-particles ensures both very high surface area and efficient light diffusion. We deposited the nc-TiO_2_ paste on alumina substrates having two parallel gold contacts, 7 mm long and spaced 0.8 mm; thickness of the film is about 1 μm. A picture of the sample is shown in Figure 2. After deposition, the films have been syntherized in two steps, 30 min each, first at 280 °C and then at 450 °C. The current-voltage characteristics of the sample showed an ohmic behaviour in the overall investigated range (0–100 V) with room temperature resistivity of the order of 2 × 10^8^ Ωm [27]. In a typical DSSCs, with a 2 μm-thick nc-TiO_2_ film, a voltage of about 0.5 V is applied and an average electric field of about 2.5 × 10^5^ V/m is settled. In our TSC measurements we therefore chose to apply a bias of 100 V across the sample, in view to get an electric field of the same order of magnitude, considering the increased distance between our planar electrodes.

To perform TSC measurements in the temperature range 5–300 K, the alumina substrates coated with the nc-TiO_2_ have been placed in a sample holder equipped with a 4 Ω wounded-wire heating resistor and a silicon temperature sensor (Leybold GmbH, Köln, Germany). The sample-holder has been inserted into a dewar containing liquid He (LHe) and positioned over the LHe vapours to ensure stable temperatures down to 4.2 K, minimize thermal inertia and reduce possible mismatch between the sample and the thermometer. Details of the experimental setup are given in [28]. Polarization of the sample and current reading was performed by a Keithley 6517 electrometer (Tektronix Ltd., Berkshire, UK), the heater was biased by a TTi QL564P power supply (TTI, Inc., Maisach-Gernlinden, Germany) and temperature was read by a DRC91C temperature controller (Lake Shore Cryotronics, Inc., Westerville, OH, USA). Priming was performed by a Light Emitting Diode (LED) source placed in front of the sample inside the sample holder. We used two priming sources: a 400 nm Ultra Violet (UV) and a 355 nm UV LED (Roithner-Lasertechnik, Vienna, Austria), having 12 mW (typical) and 8.4 mW (maximum) output power, respectively. LEDs were driven by a Systron Donner 110D pulse generator (Systron Dr, Concord, CA, USA). The light spot on the sample during illumination has a diameter of about 2 mm.

Delayed TSC measurements have been performed as follows. We primed the sample at a low temperature *T*_0_ with the LED source, biasing the sample at 100 V. Then, we waited a time interval to make fast transient effects relaxing and to get a constant temperature on the whole sample. Then, fractionated TSC analysis has been carried out performing different heating/cooling cycles up to 300 K. TSC has been also studied in the temperature range from 300 to 630 K using a different chamber where heating/cooling is performed by a system controlling temperature, pressure and gas composition. During each TSC measurement, both in the low and high temperature ranges, the scan rate was fixed at 0.1 K/s.

## 5. Experimental Results and Discussion

**Figure 3:**
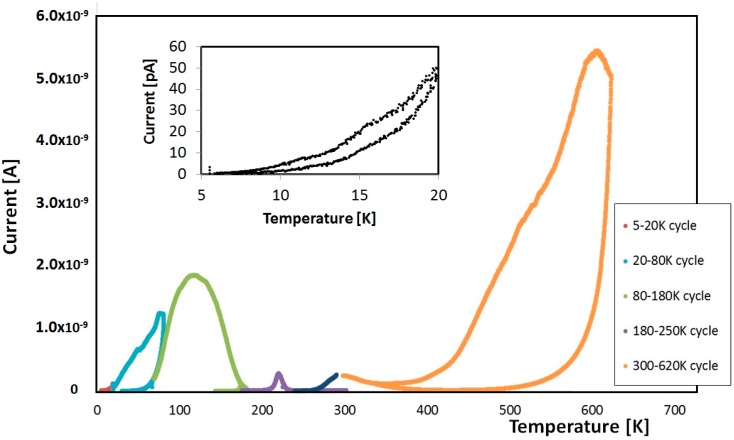
Fractionated TSC analysis performed in different cycles of measurements after ultraviolet (UV) priming at 5 K. Inset on the left shows an enlarged view of the 5–20 K temperature range.

Figure 3 shows a typical TSC spectrum observed in the overall range 5–630 K obtained with the procedure described in the previous section. A fractionated TSC is performed up to 300 K, after priming at 5 K. Then, a second priming is carried out at 300 K inside the high temperature TSC setup and a second TSC analysis is performed up to 630 K. The low temperature analysis is divided into 4 TSC fractions in the ranges: 5–20, 20–80, 80–180, 180–300 K, then, a unique TSC curve is measured after priming at 300 K up to 630 K. At last, a final cooling step from 300 to 250 K is measured to close the whole cycle.

Main conductivity processes in the two temperature ranges 5–300 K and 300–630 K are different. In fact, in the low *T* range, hopping conduction is non- negligible against free conduction. Conversely, in the high *T* range conductivity is mainly due to free carriers. Moreover, in this latter case, the influence of the surrounding gas atmosphere to the charge state of deep discrete levels in the forbidden gap cannot be neglected. Thus, in the following, we will discuss separately results measured below and above room temperature.

### 5.1. TSC Analysis below Room Temperature

**Figure 4:**
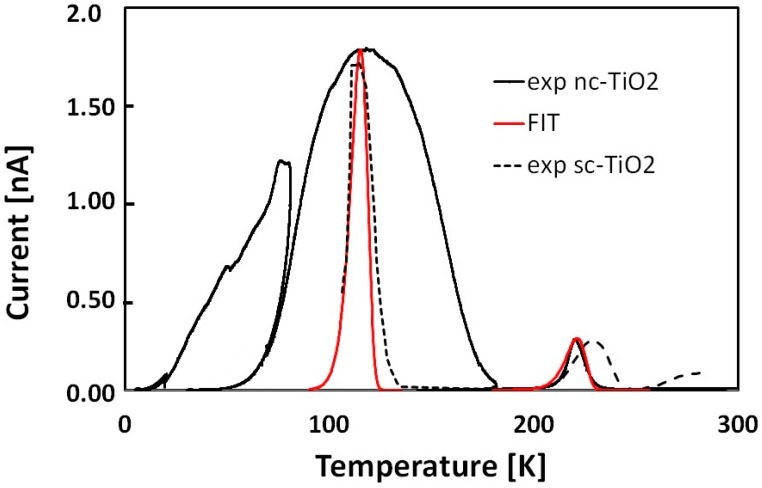
Fractional TSC measured with the nc-TiO_2_ film in the range 5–300 K compared with experimental TSC peaks reported in [29] for single crystal TiO_2_ and with a best fit obtained considering a standard TSC emission from discrete energy levels. The experimental peaks for sc-TiO_2_ have been multiplied respectively by 1700 (120 K) and 500 (230 K) to compare with those measured with the nc-TiO_2_ ones.

Figure 4 compares the TSC spectrum measured in the low *T* range together with TSC data reported in past for single crystal TiO_2_ [29]. The experimental peaks for sc-TiO_2_ have been multiplied respectively by 1700 (120 K) and 500 (230 K) to compare with those measured with the nc-TiO_2_ ones. The lower TSC signal observed in single-crystal TiO_2_ indicates that defect concentrations here is far below those encountered in the nanocrystalline morphology.

In single crystal TiO_2_, two peaks related to two discrete energy levels at 120 and 230 K are present, which can be described in terms of standard TSC emissions [26] as given in Equation (16). In nanostructured TiO_2_, we observe a much broader peak at 120 K, which cannot be described in terms of standard single-level *TSC* emission, and a peak at 220 K, rather similar to the one measured in single crystal TiO_2_. In the figure, a best fit of this latter peak is shown, obtained with a standard TSC analysis (Equation (16)), a very high capture cross section, *σ*_n_ ≈ 10^−10^ m^2^ and $E_{t}$ ≈ 0.8 eV, $N_{t}$ ≈ 10^14^ cm^−3^.

**Figure 5:**
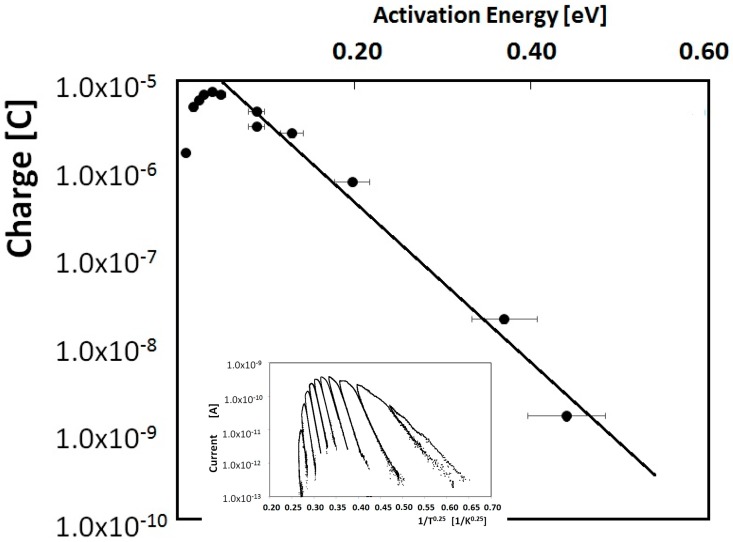
Emitted charge plotted as a function of the activation energy measured in the rising range of each peak of the fractionated TSC experiment shown in the inset. The exponential fit reflects the trend of the band tail in the deepest range within the forbidden gap. Inset: Delayed TSC in the range 5–150 K plotted as a function of 1/*T*^1/4^ to evidence the contribution of hopping conduction in the electrical transport.

To investigate the origin of the broad band peaked at 120 K, we then performed a measurement in the same temperature range with more delayed heating steps (up to 10). Results are shown in the inset of Figure 5. We note that, in a standard thermally activated emission where carriers are emitted from discrete energy levels towards the corresponding extended band, the current measured at the foot of the TSC curve in each heating/cooling step has a dependence on temperature given by: $I\left(T\right)\propto T^{2}e^{-\frac{E_{\mathrm{act}}}{K_{B}T}}$, while if hopping conduction dominates, the dependence should be (Mott’s expression): $I\left(T\right)\propto e^{-{\left(\frac{T_{0}}{T}\right)}^{4}}$ [30]. Inset of Figure 5 shows current measured in the range 5–150 K in a Mott plot. Indeed, the foot of the logarithmic plots at each heating/cooling stage is linear with *T*^−1/4^, in agreement with the fact that hopping conduction is prevailing in this temperature range and that, as suggested by [13], it should be related to a band tail deforming the DoS shape close to the mobility edge.

As described in the previous section, to investigate the DoS shape we evaluated, for each scan, the activation energy in the rising foot range of each peak and the corresponding total emitted charge. The result is shown in Figure 5, where the total charge is plotted as a function of the activation energy. Results evidence a mono-exponential DoS: $f\left(\varepsilon \right)=Q_{L}e^{\frac{\varepsilon }{E_{L}}}$(E), in the energy range 0.1–0.6 eV with $E_{L}$ tailing factor.

Best fit gives: $E_{L}$ = 47.5 meV, $Q_{L}$ = 2.8 × 10^−5^ C, values fairly in agreement with literature [31]. We note that in the low energy range of the plot, the charge is lower than the expected value indicated by the exponential trend. This can be explained considering that priming could not fill all the states in the highest part of the band tail, close to the boundary point at *ε* = 0. As a strong evidence of this, using a deeper UV LED (355 nm) the drop appears for shallower energies with respect to the shallower UV LED (400 nm). Considering a 2 mm diameter light spot and a sample thickness of about 1 μm, the effective volume involved in this process is: Vol ≈ π × 10^−12^ m^3^ and a rough estimate of the density of states is: $N_{L}=\frac{Q_{L}}{qVol}$ ≈ 5 × 10^19^ cm^−3^.

**Figure 6:**
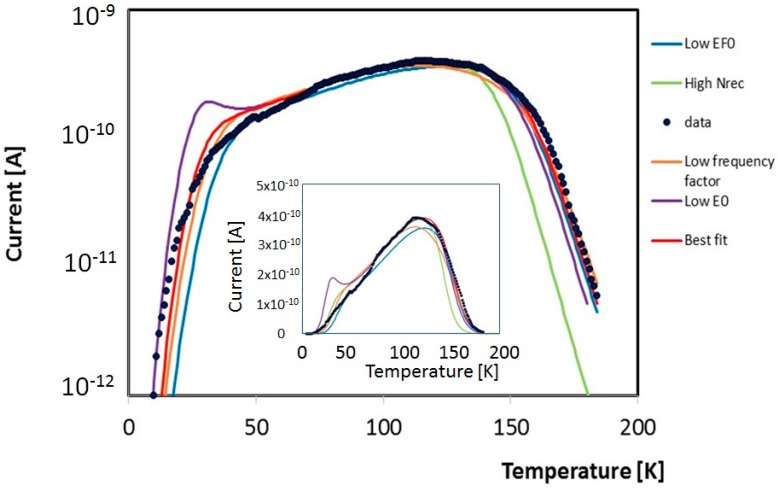
Fit procedure of data measured during a unique *TSC* emission in the range 5–200 K (convoluted from data of Figure 3). Red: best fit obtained with *N*_rec_ = 75 × 10^20^ m^−3^, frequency factor *ν*_0_ = 4 × 10^12^ s^−1^, *E*_F0_ = 66.5 meV, *E*_L_ = 60 meV, *N*_L_ = 10^20^ m^−3^. Green: same with higher concentration of recombination centres *N*_rec_ = 6 × 10^22^ m^−3^, violet: same with lower *E*_L_ = 50 meV, orange: same with lower frequency factor *ν*_0_ = 2 × 10^12^ s^−1^, blue same with lower initial Fermi level *E*_F0_ = 72.2 meV.

To best-fit our TSC measurement, we considered as fixed a group of parameters, related to the crystalline quality of our sample, mainly *N*_C0_, *μ*_C0_, *α* and *γ*, already given in [13]. Then, we determined first the best-fit of a TSC peak from a unique scan through a *χ*-square procedure by opportunely changing variable parameters starting from values of *E*_L_ and *N*_L_ obtained from Figure 5. Significant parameters as frequency factor *ν*_0_, concentration of recombination centres, *N*_rec_, initial Fermi level position *E*_F0_ (this latter due to the filling procedure and calculated from the conduction band miminum) have been opportunely changed to improve our simulated peak. Figure 6 shows, as an example, how these variable parameters can affect the process. In the figure, the red curve is our best fit, obtained with *E*_L_ = 60 meV, *N*_L_ = 10^20^ m^−3^, *N*_rec_ = 7.5 × 10^21^ m^−3^, frequency factor *ν*_0_ = 4 × 10^12^ s^−1^, *E*_F0_ = 66.5 meV. Other curves in the plot are obtained with same parameters apart from one that has been intentionally changed to evidence its influence on the simulation. Green line, uses a higher recombination centre concentration, *N*_rec_ = 6.0 × 10^22^ m^−3^, this fasten the decay of the TSC peak in the high temperature region, due to a reduced charge lifetime. Violet curve comes from a lower energy decay factor, *E*_L_ = 50 meV, enhancing emission of charges at low temperature. Orange line is characterised by a lower frequency factor, *ν*_0_ = 2 × 10^12^ s^−1^, bringing to a smoothed peak. Finally, the blue curve has been calculated using a deeper initial Fermi level, *E*_F0_ = 72.2 meV, corresponding to less initial charge within the hopping band tail after the priming process. In this case, a lower emission especially in the low temperature range is observed, as expected. So, each parameter is influencing a particular range of temperature and a peculiarity of the complex shape of the emission and, even if many parameters are inter-playing in the formation of the whole peak, each can be optimized almost individually to improve the overall simulation.

**Figure 7:**
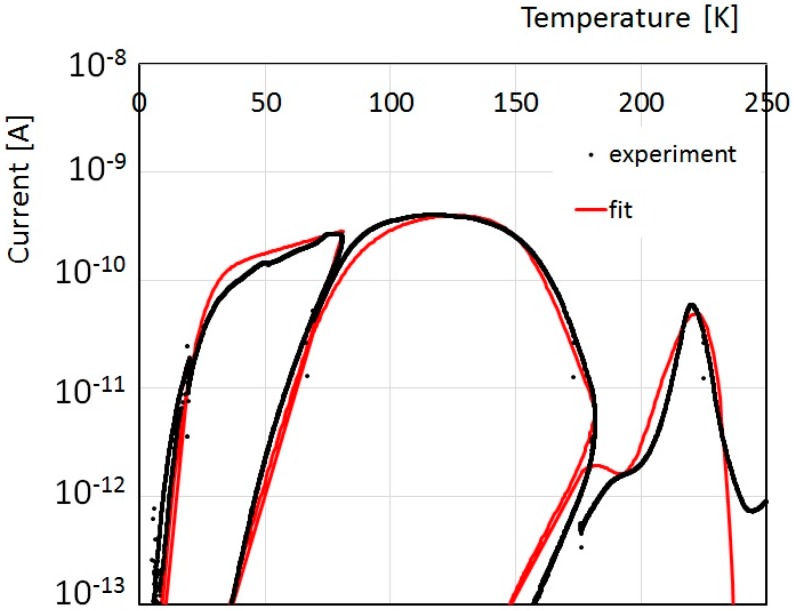
Experimental TSC measured in the 5–250 K range and best fit obtained considering the mixed conductivity model taking care of both hopping and free carriers conduction plus emission from a discrete energy level at 0.8 eV.

Best-fit of TSC experimental data shown in Figure 4 in the low temperature range, obtained using our mixed conductivity model taking care of the band tail in the range down to 0.6 eV plus a discrete level at 0.8 eV, is shown in Figure 7. Of note the agreement between numerical and experimental data in the overall range, up to almost 4 orders of magnitude of the current. A disagreement is observed at high temperature, where the experimental current stabilizes itself on the pAs range, while in the numerical model it decreases to lower values, as a consequence of the decrease of the Fermi level towards midgap. The pAs contribution to the current could be due to the residual presence of water vapor physisorbed on the film surface, as discussed in the next section.

### 5.2. TSC Analysis above Room Temperature

In this temperature range, the effect of hopping conduction should become more and more negligible against free carrier one. Moreover, physisorption and chemisorption mechanisms at surface should also participate to conduction. In particular, dangling bonds at the nc-TiO_2_ surface are capturing and releasing oxygen depending on pressure and relative humidity, these effects should be possibly investigated separately. To this purpose, we performed different sets of measurements as follows.

#### 5.2.1. Current vs. Temperature with No Priming

**Figure 8:**
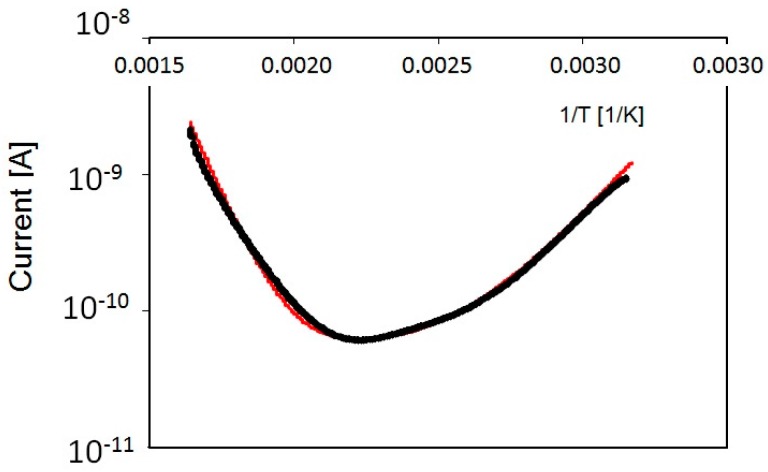
Current measured as a function of reciprocal temperature during a quasi-stationary heating process, in the range 300–600 K with no priming. Dark: data, red: best-fit.

Measurements are performed without previous priming. Using a low heating/cooling rate (0.1 K/s) as a first approximation we can assume a quasi-stationary equilibrium. The sample is kept in dark with air at a pressure of 1100 mb, slightly higher than ambient pressure. A typical measurement is presented in Figure 8.

The Arrhenius plot shows two distinct ranges: up to about 400 K the current decreases increasing the temperature *T*, then it increases with *T*. To explain this behavior we can consider the model proposed by [32], taking account of two dominant defects, one acting as a trap, the other as a recombination center (probably associated to dangling bonds at surface, releasing holes via a thermally activating process). Neglecting the small contribution of the hopping, the rate equation for the charged carriers (free electrons and holes concentrations are denoted by n,p) is then given by:

(17) $$\frac{dn}{dt}=N_{t1}c_{1}e^{-E_{t1}/K_{B}T}-Bnp-\left(n-n_{0}\right)\gamma ,$$

The first term of the right side is due to emission of electrons from the trap of energy $E_{t1}$, concentration $N_{t1}$ and frequency factor $c_{1}$. The second term describes the recombination of electrons with holes at the recombination center. We here assume that also hole capture is a thermally activated process: $p=N_{t2}c_{2}e^{-E_{t2}/K_{B}T}$, with $N_{t2}$, $E_{t2}$, $c_{2}$ respectively concentration, energy and frequency factor of the recombination center and B a probability coefficient. The third term in Equation (17) takes account of other possible free electron removal mechanisms, with a coefficient γ, as trapping from deeper levels, acting on the excess concentration, *n*_0_ being the equilibrium electron concentration.

During the current temperature measurements in dry fluxed air the system is actually in a quasi-stationary regime, so we can reasonably consider $\frac{dn}{dt}=0$, then:

(18) $$n=\frac{n_{0}+\frac{N_{t1}c_{1}}{\gamma }e^{-\frac{E_{t1}}{K_{B}T}}}{1+\frac{BN_{t2}c_{2}}{\gamma }e^{-\frac{E_{t2}}{K_{B}T}}}$$

which gives a current dependence with temperature as:

(19) $$I_{\mathrm{fit}}=F\left(\frac{a+be^{-\frac{E_{t1}}{K_{B}T}}}{1+de^{-\frac{E_{t2}}{K_{B}T}}}\right)$$

with $a=q\Sigma \mu_{C}n_{0};\;b=q\Sigma \mu_{C}\frac{N_{t1}c_{1}}{\gamma };\;d=\frac{BN_{t2}c_{2}}{\gamma }$ and where again Σ is the surface normal to electric field *F*. Best-fit of our data with Equation (19), shown in Figure 8, is obtained with energy values: $E_{t1}$ = 1.30 ± 0.05 eV and $E_{t2}$ = 0.40 ± 0.05 eV. To briefly comment on these values, we observe that e.g., in [33] shallower defect levels at ∼0.24−0.4 eV in anatase TiO_2_ were attributed to Ti interstitials, while deeper ones at ∼0.9−1.1 eV to oxygen-vacancies.

#### 5.2.2. *TSC* after Storage in Dark and Humid Environment

**Figure 9:**
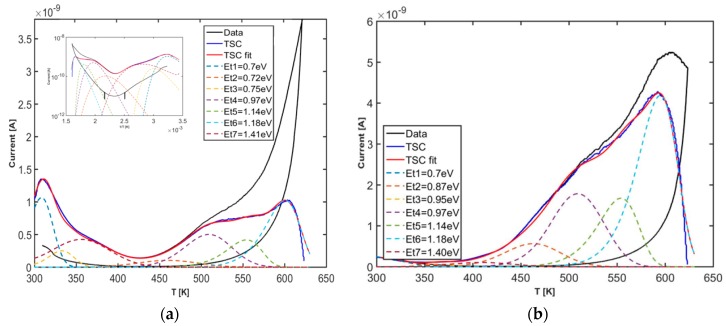
TSC after storage in ambient air (*T* = 300 K) in humid environment (*rh* = 20%) for (**a**) two days (inset: logarithmic plot) experimental data (black); TSC with background subtracted (blue); TSC best fit (red curve) obtained with a set of 7 peaks with energy *E*_t0_ given in the legend; (**b**) TSC measurements after four days storage and best fit with same *E*_t0_ parameters as in (**a**).

To analyze the effect of water vapor on TSC data, we first primed the sample by keeping it in a controlled humid environment (*rh* = 20%) in dark at room temperature (T = 300 K) for selected time intervals, up to 14 days. Then, a TSC heating/cooling cycle has been performed by fluxing dry air with a pressure slightly higher than atmosphere, in view to measure only emissions originated during charging in the storage period. As an example, Figure 9a,b show TSC experimental data obtained after 2 and 4 days storage respectively. Curves labeled “data” report experimental measurements, while those labeled TSC show the thermally stimulated currents obtained by subtracting the current measured during the cooling scan from that measured during the heating scan. TSC emissions are observed within two distinct ranges of temperatures: one from ambient temperature, up to 400 K, the other one above 400 K.

An evaluation of the main TSC components involved in these measurements has been carried out in order to identify the origin of the emissions. Measurements show statistically broadened emissions They can be fitted using TSC peaks convoluted with a gaussian as given in Equation (13). Best fits have been obtained by opportunely changing trap concentration $N_{t}$ for a same set of ($E_{t}$, $\sigma_{E_{t}},\;\sigma_{n}$), within errors, best fitting the two measurements. Up to seven energy levels are required to fit our data. Parameters are shown in Table 1, energy levels are peaked at $E_{\mathrm{to}}=$ 0.7–1.14 eV and are characterized by an energy spread $\sigma_{E_{t}}$ up to 70 meV. As a general trend, increasing the storage time, peaks at low temperatures decrease their concentration $N_{t}$, while those at high temperatures increase *N*_t_. A source of uncertainty in the determination of concentration for the peak at ambient temperature is due to the increasing background current observed during the cooling stage, observed especially in Figure 9a. This effect can indicate reversible charging/discharging of the involved energy states, maybe due to adsorbing/desorbing from the porous alumina substrate.

To comment on the origin of these peaks, we observe that our measurements are in agreement e.g., with temperature programmed desorption (TPD) analyses measured in past with TiO_2_ after exposure to water. Four peaks at 155, 190, 295, and 490–540 K were observed by [34,35,36,37], the first three assigned to molecular desorption from multilayer, second layer, and first layer states, while the higher temperature feature was assigned to recombinative desorption. Effusion peaks from water were also observed in [34] by thermal desorption in the 150–350 K range from porous nanostructured TiO_2_ and attributed to physiosorbed H_2_O, while in [35], two H_2_O effusion peaks were detected around 440 and 650 K. Moreover, physically adsorbed and dissociated H_2_O molecules in nanostructured anatase TiO_2_ have been studied by Fourier Transform InfraRed (FTIR) emission spectroscopy at different temperatures in the range 100–300 °C [38]. A 3665 cm^−1^ band assigned to OH hydrogen bonded (adjacent) OH groups was observed to considerably decrease when the sample was heated from 373 to 573 K, while one at 3705 cm^−1^, attributed to isolated OH groups, more difficult to remove from the surface than adjacent OH groups, only slightly changed. A 3250 cm^−1^ component attributed to the stretching vibration of water molecules that are hydrogen bonded was considerably weakened when heated up to 423 K, while the one at 3400 cm^−1^, related to hydrogen-bound surface OH groups (Ti OH), became visible at this temperature. This latter and the 1625 cm^−1^ band (identified as the water bond-bending vibration mode) finally disappeared at 573 K.

Although a direct correlation between TPD, FTIR and our TSC measurements should be performed when same kind of samples and same experimental conditions are considered, we can qualitatively conclude that TSC peaks in the range up to 400 K should be related to adsorbed molecular water, while dissociated species, as hydrogen-bonded OH groups, should be involved in emissions in the range 400–600 K. In our measurements, increasing the storage time, peaks at low temperature are decreasing, while those at high temperature are increasing, and eventually saturating. This can be explained considering that molecular adsorbed water, in time, is slowly evolving into the formation of hydrogen bonded OH species, more stable at room temperature.

#### 5.2.3. TSC after Illumination in He Atmosphere with Different Pressures

To evidence the effect on TSC of the oxygen-exchange at surface, we performed a set of measurements were the sample was primed in a dry He atmosphere at different pressure, from 10^−6^ to 1 bar, at room temperature (*T* = 300 K), during illumination with a Xe lamp for a selected time interval. Then, TSC heating/cooling cycles were carried out by fluxing dry air with a pressure slightly higher than atmosphere, to measure only emissions originated during charging in the storage period. In fact, it is known that oxygen vacancies can be created by annealing TiO_2_ at elevated temperatures in an oxygen-poor environment, such as a pure He gas atmosphere or vacuum condition [39].

**Figure 10:**
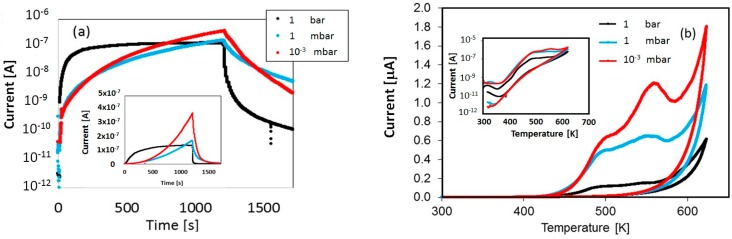
(**a**) Photocurrent measured during priming by illuminating with a Xe lamp the nc-TiO_2_ sample in an He atmosphere of different pressures at *T* = 300 K; (**b**) TSC measured after priming with 1 bar,1 mbar, 10^−3^ mbar. Heating/cooling cycles are performed in He atmosphere with 1 bar pressure.

Results of photocurrent measurements during priming are shown in Figure 10a. TSC curves after priming in these conditions are shown in Figure 10b.

**Figure 11:**
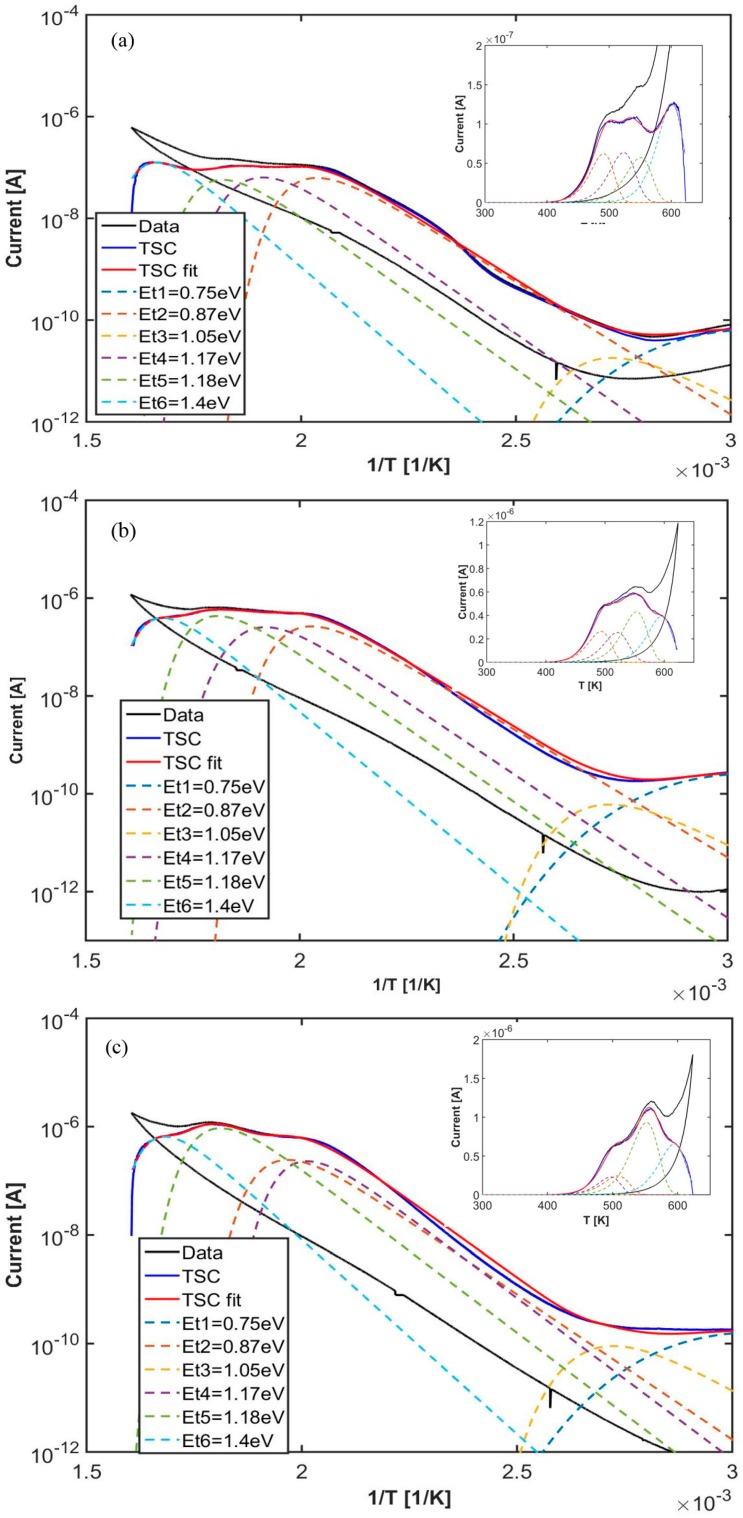
TSC response after priming in He atmosphere with a pressure in chamber (**a**) 1 bar; (**b**) 10^−3^ bar (**c**) 10^−6^ bar. TSC experimental data (black); Blue: TSC heating scan subtracted from background (cooling stage); dotted curves: TSC peaks convoluted with a gaussian, parameters are given in Table 2.

While at 1 bar the photocurrent is almost saturating during priming, at low pressure it increases superlinearly, a fact that can be explained considering the creation of extra oxygen-vacancies, which are releasing an ever increasing free carriers concentration, so favoring the passivation of deep traps during priming. To comment on TSC curves reported in Figure 10b (inset: logarithmic plot) we observe that, in the case of vacuum priming, the tail in the cooling stage of increasing current below 400 K observed in case of humid environment (Figure 8 and Figure 9) is almost absent. Then, higher TSC emissions are observed in the high temperature range in vacuum, showing an increasing number of passivated deep traps in the priming stage. Best fits of the TSC measurements in He atmosphere have been performed starting with the same set of energy levels used in the previous section. They are shown in Figure 11a–c. Measured emissions have been calculated considering TSC peaks statistically broadened as given in Equation (13). Results are shown in Figure 11a–c respectively for the cases of 1, 10^−3^, 10^−6^ bar. Best-fit procedure turns out in a six-fold emission, with trap parameters listed in Table 2. The fit has been performed considering the same set of values ($E_{t}$, $\sigma_{E_{t}},\;\sigma_{n}$), within errors, for the three measurements, and best-fitting the TSC scans obtained by subtracting the response during cooling to the one measured during heating and by opportunely changing the trap concentration values $N_{t}$. Trap parameters given in Table 2 are in agreement with the model in [40] indicating that localized donor states originating from oxygen vacancies are located at 0.75–1.18 eV below the conduction band of Titania.

The six peaks used to best-fit our TSC curves are characterized by the same ($E_{t}$, $\sigma_{Et},\;\sigma_{n}$), within errors, used to fit TSC measurements in the humid environment (apart of the shallowest two levels that here are present in only one component). Here, at every pressure analyzed, shallowest levels have negligible concentrations with respect to deepest levels. Looking to plots in Figure 11, we observe a good agreement between fit and data on a four-orders of magnitude scale. Logarithmic plots are shown as a function of 1/T: the observed linear trend is in favour with our previous observation, that hopping conduction in this high-temperature range is negligible.

To comment on these measurements, we observe that in low pressure/high temperature conditions the probability to form oxygen-vacancies on the surface of the nanostructured titania increases, an evidence widely discussed, e.g., in [41]. It is thus reasonable to hypothesize that TSC peaks shown in Figure 9, Figure 10 and Figure 11 are related to such a phenomenon, as oxygen-atoms released from the surface leave behind electrons in the conduction band which are collected at electrodes and participate to the emission process. The fact that defects related to these TSC peaks are the same measured after exposure to humid environment, (apart from the shallowest levels which we attributed to adsorbed molecular water) evidences that also OH groups are more likely to be associated to oxygen vacancies, as e.g., suggested in [41].

## 6. Conclusions

Nanocrystalline Titanium dioxide is widely applied as a high gap semiconducting material in many optoelectronic devices, from solar cells to gas sensors, where its peculiar electronic properties and high chemical reactivity play a crucial role. To attain good performances in terms of efficiency and photoactivity, Titania is mostly used in form of porous nanocrystalline thin films, a material characterized by a high degree of microstructural disorder, which detrimental effect in transport properties should be taken in great care and possibly minimized.

The thermally stimulated current technique is one of the most effective tool for characterizing electrical defects in semiconductors. In this paper, we have used this method to get an overall picture of the defect distribution in nanocrystalline Titania used in state-of-art DSSC devices. The model of TSC used to interpret our experimental results, briefly described in this work, takes account of the heavily disordered microscopic nature of nc-TiO_2_. Mixed conductivity with non-negligible contributions from hopping between localized defects grouped in a band-tail below the conduction band is considered. Moreover, a broadening of the energy levels associated to discrete defects in the forbidden gap, has been accounted for, by convoluting the TSC standard emission with a gaussian distribution. Shallow-to deep energy levels ranging from 0.1 to 1.4 eV have been studied via a thermal spectroscopy spanning from 5 to 630 K and interpreted with this model: main results of our analysis can be summarized as follows.

An exponential DoS tail within the forbidden gap has been observed in the range 0.1–0.6 eV from the bottom of the conduction band, with energy tailing factor of 50–60 meV, characterized by density of states of the order of 1 × 10^20^ cm^−3^. This tail is responsible for a large TSC emission visible after priming with a UV source at 5 K for temperatures up to approximately 150 K. At higher temperatures, up to room temperature and above, the hopping contribution to conduction becomes more and more negligible against free carrier one and contributions from discrete energy levels emitting in the conduction band become visible. Similar to single crystal TiO_2_, a sharp TSC peak at 220 K is observed, with energy 0.8 eV, probably related to water adsorption. Above room temperature, dark current measured as a function of the temperature without any priming reveals to be non-negligible, as it should be due in pure intrinsic TiO_2_ material. So, we studied it separately before any TSC analysis. Measurements as a function of temperature shows a double exponential trend, with a minimum at about 400 K. To explain this behavior a model taking account of two dominant defects, one acting as a recombination center, the other as a trap has been considered. The activation energy measured in this experiment in dark, for the recombination center, is about 0.4 eV a value compatible with dangling bonds at surface [22]. The trap energy, evaluated as about 1.3 eV, is in the highest energy range found for trap states observed in our further analyses.

Then, TSC response above background current has been studied as a function of the temperature, as a spontaneous emission observed after a prolonged storage of the sample in moderately (*rh* = 20%) humid ambient air. They are best-fitted using gaussian-broadened TSC emissions, with average energy 0.7–1.4 eV, characterised by uncertainties up to 70 meV. Two groups of peaks are measured, respectively below and above temperature. Peaks up to 350 K are probably related to molecular desorption from multilayer, second layer, and first layer states, while the higher temperature features should be assigned to desorption of OH groups [42]. Our measurements show that increasing the storage time molecolar adsorbed water slowly evolves into recombinative species. Finally, to evidence the relationship between TSC emissions and vacancy-oxygen defects, measurements have been carried out after priming in inert atmosphere (He) at different pressures. Main TSC emissions, observed in the high temperature range, 400–630 K, are best-fitted considering energy levels for localized energy states in the range 0.75–1.18 eV, in agreement with a model from [41] for donor states related to oxygen-vacancies below the conduction band.

## Figures and Tables

**Table 1:** Best fit trap parameters of the *TSC* measured after storage in humid air environment at room temperature.

Peak #	*E*_t_ [eV]	*σ*_t_ [eV]	*σ*_n_ [cm^2^]	*N*_t_ [10^16^ cm^−3^] 2 Days	*N*_t_ [10^16^ cm^−3^] 4 Days
1–3	0.70–75	0.070	10^−20^–10^−18^	0.34	0.06
4	0.97	0.070	4 × 10^−20^	0.42	0.07
5	1.14	0.020	2 × 10^−19^	0.3	1.43
6	1.18	0.007	5 × 10^−20^	0.82	1.15
7	1.41	0.007	7 × 10^−19^	0.25	1.14

**Table 2:** Best fit trap parameters of the TSC measured after storage in different pressures of He atmosphere at room temperature during illumination with a Xe lamp.

Peak #	*E*_t_ [eV]	*σ*_t_ [eV]	*σ*_n_ [cm^2^]	*N*_t_ [10^18^ cm^−3^] 1 Bar	*N*_t_ [10^18^ cm^−3^] 10^−3^ Bar	*N*_t_ [10^18^ cm^−3^] 10^−6^ Bar
1	0.750	0.040	1 × 10^−18^	1.8 × 10^−4^	0.7 × 10^−3^	0.5 × 10^−3^
2	0.870	0.001	3 × 10^−18^	0.4 × 10^−4^	0.2 × 10^−3^	0.3 × 10^−3^
3	1.031	0.015	3 × 10^−20^	0.31	1.35	1.35
4	1.166	0.030	1 × 10^−18^	0.37	1.53	1.08
5	1.177	0.007	5 × 10^−20^	0.38	2.87	6.21
6	1.411	0.009	7 × 10^−19^	0.94	2.88	4.77
